# T Wave Amplitude Correction of QT Interval Variability for Improved Repolarization Lability Measurement

**DOI:** 10.3389/fphys.2016.00216

**Published:** 2016-06-07

**Authors:** Martin Schmidt, Mathias Baumert, Hagen Malberg, Sebastian Zaunseder

**Affiliations:** ^1^Institute of Biomedical Engineering, Technische Universität DresdenDresden, Germany; ^2^School of Electrical and Electronic Engineering, The University of AdelaideAdelaide, SA, Australia

**Keywords:** ECG, QT interval variability, T wave amplitude, risk stratification, DEFINITE

## Abstract

**Objectives:** The inverse relationship between QT interval variability (QTV) and T wave amplitude potentially confounds QT variability assessment. We quantified the influence of the T wave amplitude on QTV in a comprehensive dataset and devised a correction formula.

**Methods:** Three ECG datasets of healthy subjects were analyzed to model the relationship between T wave amplitude and QTV. To derive a generally valid correction formula, linear regression analysis was used. The proposed correction formula was applied to patients enrolled in the Evaluation of Defibrillator in Non-Ischemic Cardiomyopathy Treatment Evaluation trial (DEFINITE) to assess the prognostic significance of QTV for all-cause mortality in patients with non-ischemic dilated cardiomyopathy.

**Results:** A strong inverse relationship between T wave amplitude and QTV was demonstrated, both in healthy subjects (*R*^2^ = 0.68, *p* < 0.001) and DEFINITE patients (*R*^2^ = 0.20, *p* < 0.001). Applying the T wave amplitude correction to QTV achieved 2.5-times better group discrimination between patients enrolled in the DEFINITE study and healthy subjects. Kaplan-Meier estimator analysis showed that T wave amplitude corrected QTVi is inversely related to survival (*p* < 0.01) and a significant predictor of all-cause mortality.

**Conclusion:** We have proposed a simple correction formula for improved QTV assessment. Using this correction, predictive value of QTV for all-cause mortality in patients with non-ischemic cardiomyopathy has been demonstrated.

## 1. Introduction

Beat-to-beat fluctuations of ventricular repolarization are reflected in the QT interval variations of surface ECG. Several studies have shown promise in exploiting QT interval variability (QTV) for quantifying temporal repolarization lability (Baumert et al., 2016a) and associations between elevated QTV and cardiac mortality have been reported (Berger et al., 1997; Piccirillo et al., 2007).

However, measuring the rather subtle beat-to-beat changes in QT interval remains challenging. Novel techniques that utilize templates have been proposed and demonstrated robustness toward common ECG artefacts and noise (Berger et al., 1997; Starc and Schlegel, 2006; Schmidt et al., 2014b). The relationship between QTV and ECG artefacts (Baumert et al., 2012; Schmidt et al., 2014b) and sampling rate (Baumert et al., 2016b) has been investigated and recommendations have been made (Baumert et al., 2016a). In spite of that, QTV analyses often show indifferent results in conditions where increased repolarization variability is anticipated (Baumert et al., 2012). A potential reason for this discrepancy is the negative correlation between QTV and T wave amplitude, which is particularly evident when T waves are small and the signal-no-noise ratio is low (Baumert et al., 2016a). Consequently, differences in the T wave amplitude across subjects and/or ECG leads may confound statistical analyses of QTV and impede its clinical utility (Baumert et al., 2016a).

The presented study was conducted to (1) quantify the influence of the T wave amplitude on QTV in a comprehensive dataset and (2) devise a correction formula to account for the influence of T wave amplitude on QTV.

## 2. Materials and methods

### 2.1. Data

To assess the relationship between T wave amplitude and QTV quantitatively and subsequently to develop a correction formula, three ECG datasets of healthy subjects were analyzed:

Technical details for all datasets are listed in Table 1.

**Table 1:** **Descriptions and technical details of datasets used in this study**.

**Dataset [Abbr.]**	**Medical condition**	**Description and technical details (Sampling rate and lead configuration)**
Athletic dataset [ATHLETE] 20 records (20–30 min)	Healthy	Athletes (lying and standing) 1600 Hz, modified 3-Lead Frank ECG
PTB Diagnostic ECG Database [PTB] 69 records (2 min)	Healthy	Healthy subjects of PTB Diagnostic ECG Database 1000 Hz, Standard 12-Lead + 3-Lead Frank ECG
E-HOL-12-0140-008 [TQT] 140 records (60 min)	Healthy	Baseline of E-HOL-12-0140-008 – Thorough QT Study # 2 1000 Hz, 12-Lead Holter ECG
E-HOL-03-0401-017 [DEFINITE] 393 records (24 h)	Non-ischemic cardiomyopathy	E-HOL-03-0401-017 – DEFINITE 500 Hz, 3-Lead Frank Holter ECG

Athlete dataset [ATHLETE]: The dataset consists of 20 recordings from 10 healthy athletes (5 females, age: 24.8 years [24.7−26.4]; 5 males, age: 26.6 years [26.5−28.8]); 3-lead ECG (modified Frank lead system) (Baumert et al., 2006). ECG were recorded during rest in the supine position for 30 min and subsequently during standing for another 20 min. Because of varying number of available leads (up to three leads), we used the lead with the highest T wave amplitude (Baumert et al., 2016a) for further analyses.

PTB Diagnostic ECG Database [PTB]: The dataset consists of 69 healthy control subjects (17 females, age: 42 ± 18 years; 52 males, age: 40±13 years) included in the Physikalisch-Technische Bundesanstalt (PTB) diagnostic ECG database (Bousseljot et al., 2009). Standard 12-lead ECGs were recorded during rest for about 2 min.

Thorough QT Study dataset [TQT]: The dataset comprises ECG of 72 healthy subjects (29 females, age: 47 ± 8 years; 43 males, age: 40±11 years) of the E-HOL-12-0140-008 dataset from the Telemetric and Holter ECG Warehouse (THEW) repository; 60 min baseline parts (before treatment dosing) of standard 24h-Holter 12-lead ECGs were analyzed.

To investigate the efficiency of the proposed T wave amplitude correction, we analyzed the predictive value of QTV for all-cause mortality in patients with non-ischemic dilated cardiomyopathy enrolled in the Evaluation of Defibrillator in Non-Ischemic Cardiomyopathy Treatment Evaluation trial (DEFINITE): Therefore, Holter ECG of 236 patients (67 females, age 60 ± 14 years; 168 males, age: 58 ± 12 years; 1 not specified) included in the E-HOL-03-0401-017 dataset [Telemetric and Holter ECG Warehouse (THEW)] were considered. All patients had a left ventricular ejection fraction < 36% and were randomized to receiving standard medical therapy with or without an ICD (Kadish et al., 2004). 24h-Holter 3-lead (Frank lead system) ECGs were performed at enrollment and after up to 5 years' follow-up. The all-cause mortality during the follow-up period was 4.8%. In total the dataset consists of 393 recordings. Because of highest amplitude of T wave (Baumert et al., 2016a), pseudo-orthogonal lead bipolar Z was used for QT interval analysis.

### 2.2. QT interval measurement

#### 2.2.1. ECG pre-processing

A high pass filter (cut-off frequency 0.3 Hz) was applied to all raw ECGs. Subsequently, QRS complexes were detected using the algorithm described by Afonso et al. (1999) and included in the biosig toolbox (Vidaurre et al., 2011).

#### 2.2.2. QT delineation

We used two different algorithms for QTV measurement to verify the T wave amplitude – QTV relationship:

(1) 2DSW

This algorithm employs a template matching technique that relies on two-dimensional signal warping (2DSW; Schmidt et al., 2014b). The 2DSW algorithm was shown to deliver accurate and robust results even in noisy conditions (Zaunseder et al., 2014). It is able to account for complex morphological changes in the ECG waveform by adopting an averaged template beat. To track common features of interest, e.g., the P, QRS, or QT intervals or amplitude information, the template is annotated in a semi-automated fashion (Laguna et al., 1994). The template adaption is realized by superimposing a 2D mesh of warping points on the template beat. Each warping point is shifted in time- and amplitude-direction to minimize the Euclidean distance between the template beat and the beat under consideration. By applying this technique to all incoming beats, beat-to-beat changes of common time and amplitude ECG features can be measured and used for a comprehensive ECG variability analysis.

We applied automated beat rejection to exclude unusable heart beats from further QTV analysis [rejection criterion (Schmidt et al., 2014b) was set to 10% of the median T wave amplitude ${\bar{T}}_{amp}$].

(2) Template stretching

The template stretching technique has been proposed by Berger et al. to measure beat-to-beat QT interval changes (Berger et al., 1997). The main idea is to define a template QT interval and mark the beginning of the QRS complex and the end of the T wave (To ensure comparable results, we used the same annotations as in 2DSW templates). The adaption of the template to all incoming beats is realized by linear stretching or compressing of the template in time that it matches best. QT interval measurement is possible with the QT template reference and the stretching factor.

### 2.3. QTV quantification

Extraction and identification of time (*X*) and amplitude (*Y*) coordinates relating to the end of the P wave (*Pend*), Q wave (*Qonset*), the peak of the T wave (*Tpeak*), and its end (*Tend*) of every beat *i* is necessary for calculating QT interval *QT*_*i*_, iso-electric level (*Isolevel*), and T wave amplitude *T*_*i, amp*_ [the *Isolevel*_*i*_ is defined as median value of the “flattest” waveform segment (20 ms) between *X*_*i, Pend*_ and *X*_*i, Q*_ (Jager et al., 2003)]:

$$\begin{array}{l} \;QT_{i}=X_{i,Tend}-X_{i,Qonset}\\ T_{i,amp}=Y_{i,Tpeak}-Isolevel_{i} \end{array}$$

We used the standard deviation of QT intervals (SDQT) to quantify QTV and the median of the absolute T wave amplitude (${\bar{T}}_{amp}$) to approximate T wave amplitude for a defined interval of *i* = 1, 2, …, *N* beats:

$$\begin{array}{l} SDQT=\sqrt{\frac{1}{N-1}\sum_{i=1}^{N}{\left|QT_{i}-\bar{QT}\right|}^{2}}\\ \;{\bar{T}}_{amp}=median(\left|T_{amp}\right|) \end{array}$$

### 2.4. Modeling of the T wave amplitude – QTV relationship

Based on previous observations (Hasan et al., 2012) a double logarithmic model was imposed. Linear regression analyses were conducted to specify the model parameters of the log-transformed data:

$$\log_{10}(SDQT)=m_{c}\cdot \log_{10}({\bar{T}}_{amp})+n_{c}$$

where *m*_*c*_ is the slope of the linear regression function and *n*_*c*_ the intercept (normalization coefficient).

To eliminate the T wave amplitude dependency of QTV, logarithmic subtraction was used. The T wave amplitude corrected QT interval standard deviation (cSDQT) can then be defined as:

$$\begin{array}{l} \log_{10}(cSDQT)=\log_{10}(SDQT)-m_{c}\cdot \log_{10}({\bar{T}}_{amp})+n_{c}\\ \;cSDQT=SDQT\cdot 10^{\left(m_{c}\cdot \log_{10}\left(\frac{{\hat{T}}_{amp}}{{\bar{T}}_{amp}}\right)\right)} \end{array}$$

where ${\hat{T}}_{amp}$ is the T wave amplitude normalization coefficient and $n_{c}=m_{c}\cdot \log_{10}\left({\hat{T}}_{amp}\right)$.

Accordingly, the so-called QT interval variability index (QTVi) that was introduced by Berger et al. (1997) as a non-invasive measure of repolarization lability was used, but utilizing RR intervals rather than heart rates (Piccirillo et al., 2001):

$$QTVi=\log_{10}\left(\frac{\frac{SDQT^{2}}{QT_{mean}{}^{2}}}{\frac{SDRR^{2}}{RR_{mean}{}^{2}}}\right),$$

where *RR*_*mean*_ is the mean of RR time series, SDRR the standard deviation of RR time series and *QT*_*mean*_ the mean of QT interval series, can be corrected for the T wave amplitude (cQTVi):

$$cQTVi=\log_{10}\left(\frac{\frac{cSDQT^{2}}{QT_{mean}{}^{2}}}{\frac{SDRR^{2}}{RR_{mean}{}^{2}}}\right).$$

After some rearranging it can be rewritten as:

$$cQTVi=QTVi+2\cdot m_{c}\cdot \log_{10}\left(\frac{{\hat{T}}_{amp}}{{\bar{T}}_{amp}}\right)$$

## 3. Results

### 3.1. Identification of the model parameters

#### 3.1.1. Comparison of healthy subjects

**Figure 1:**
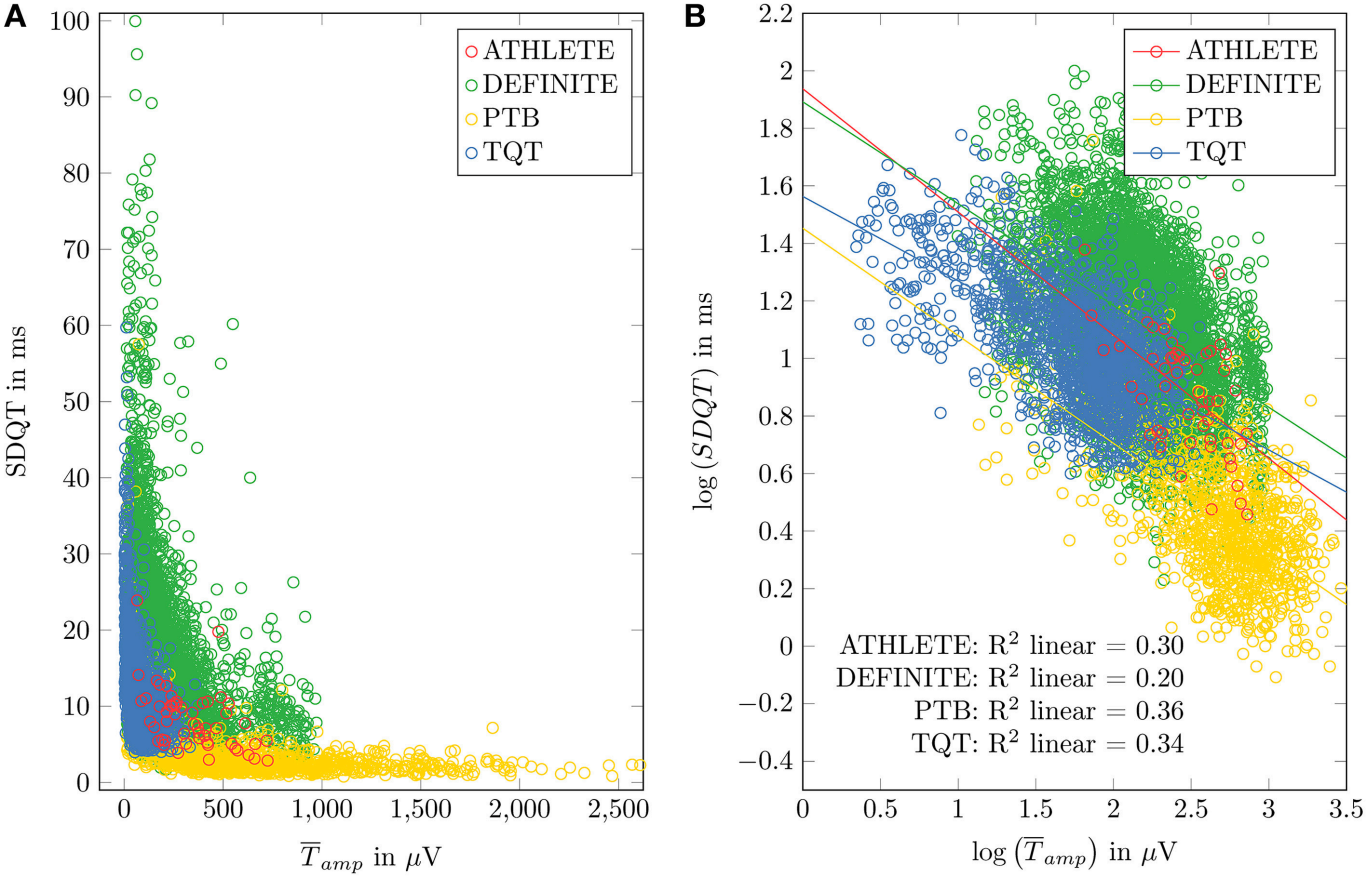
**Relation between SDQT and T wave amplitude (${\bar{T}}_{amp}$) in datasets of healthy subjects (ATHLETE, PTB and sTQT) and DEFINITE dataset is shown in (A)**. Linear regression analysis applied to logarithmized SDQT and logarithmized ${\bar{T}}_{amp}$ is plotted in **(B)**.

ECG data from healthy subjects were used to identify the regression coefficient of the model. Figure 1 shows scatter plots and regression functions of three different datasets (ATHLETE, PTB, and TQT) of healthy subjects. Significant correlation between SDQT and ${\bar{T}}_{amp}$ (Pearson correlation coefficient *r* = 0.54, *p* < 0.001) and between logarithmic SDQT (log_10_(*SDQT*)) and logarithmic T wave amplitude ($\log_{10}\left({\bar{T}}_{amp}\right)$; Pearson correlation coefficient *r* = 0.83, *p* < 0.001) were observed in all healthy subjects. For detailed results see Table 2.

**Table 2:** **Results of correlation and regression analysis for all datasets and QT interval extraction algorithms**.

**Dataset**		**QT interval extraction algorithm**	**Pearson correlation coefficient *r***		**Linear regression slope *m_c_***
			**SDQT and ${\bar{T}}_{amp}$**	**log_10_(*SDQT*) and ${\text{log}}_{10}\left({\bar{T}}_{amp}\right)$**	**log_10_(*SDQT*) and ${\text{log}}_{10}\left({\bar{T}}_{amp}\right)$**
Healthy	ALL	2DSW	0.537^***^	0.826^***^	−0.501^***^
		Template stretching	0.246^***^	0.738^***^	−0.454^***^
	ATHLETE	2DSW	0.471^**^	0.544^***^	−0.427^***^
		Template stretching	0.193	0.293^*^	−0.270^*^
	PTB	2DSW	0.338^***^	0.598^***^	−0.374^***^
		Template stretching	0.090^**^	0.531^***^	−0.393^***^
	TQT	2DSW	0.456^***^	0.584^***^	−0.293^***^
		Template stretching	0.159^***^	0.241^***^	−0.103^***^
	DEFINITE	2DSW	0.333^***^	0.449^***^	−0.354^***^

*Note that data set size differs (see Table 1) and therefore quantitative comparability of correlation coefficients is limited*.

* p < 0.05;

** p < 0.01;

*** p < 0.001;

*SDQT, standard deviation of QT intervals; ${\bar{T}}_{amp}$, median T wave amplitude*.

To obtain a general slope that is not specific to the datasets' variations in means of SDQT and ${\bar{T}}_{amp}$, linear regression slopes of the three datasets of healthy subjects (ATHLETE, PTB, and TQT) were averaged, resulting in

$$m_{c}=-0.36\;\frac{ms}{\mu V}.$$

A T wave amplitude normalization was introduced to allow for inter-dataset comparisons. We used the mean T wave amplitude ${\hat{T}}_{amp}$, calculated by averaging ${\bar{T}}_{amp}$ of all analyzed healthy records (lead independent), resulting in:

$${\hat{T}}_{amp}=300\;\mu V.$$

### 3.2. Influence of QT interval extraction method

Comparing 2DSW with the template stretching algorithm shows similar results regarding the correlation and regression between SDQT and ${\bar{T}}_{amp}$ and between log_10_(*SDQT*) and $\log_{10}\left({\bar{T}}_{amp}\right)$ (For details see Table 2).

Because the standard implementation of the template stretching method is limited to QT interval extraction and does not provide T wave amplitude values, T wave amplitudes extracted by 2DSW have been used for correction for the further analysis.

### 3.3. Between-lead comparisons of the regression coefficient

ECG lead dependent analysis showed similar regression slopes (*m*_*c*_) for multichannel datasets: PTB: *m*_*c, PTB*_ = −0.40 ± 0.09 [mean regression slopes of all leads where a significant relation was found (Student's *t*-test; *p* < 0.05)] and dataset TQT: *m*_*c, QT*_ = −0.33 ± 0.05 (12 of 12 leads, where *p* < 0.05).

### 3.4. Influence of T wave amplitude correction on QTV assessment

#### 3.4.1. Effect of orthostatic stress on QTV (ATHLETE dataset)

The group average of ${\bar{T}}_{amp}$ [*p* < 0.05 (Student's *t*-test)] was significantly higher in the supine position (398 ± 182 μ*V*) compared to standing (290 ± 150 μ*V*). Conversely, SDQT was significantly lower in the supine position compared to standing (5.88 ± 2.23 *ms* vs. 11.09 ± 3.70 *ms*; *p* < 0.001). When correcting for T wave amplitude, cSDQT was significantly lower while lying compared to standing (5.88 ± 2.23 *ms* vs. 11.09 ± 3.70 *ms*; *p* < 0.001). No notable differences in QTV were observed in corrected vs. uncorrected data.

#### 3.4.2. Predictive value of QTV for all-cause mortality in non-ischemic cardiomyopathy (definite trial)

**Figure 2:**
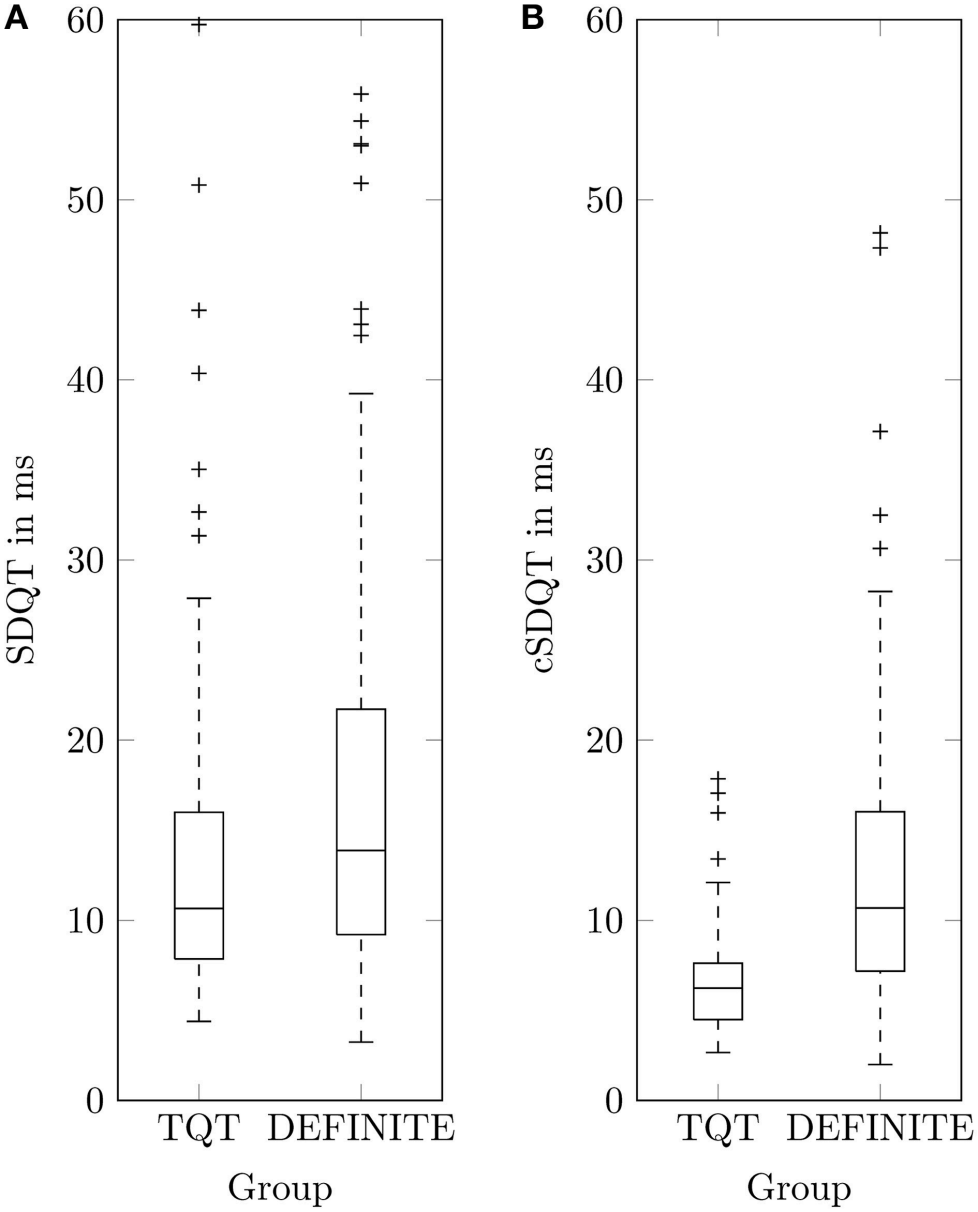
**Boxplots of QT standard deviation SDQT (A) and corrected QT standard deviation cSDQT (B) for healthy TQT dataset and patients with non-ischemic cardiomyopathy (DEFINITE)**.

Linear regression analysis of log_10_(*SDQT*) and $\log_{10}\left({\bar{T}}_{amp}\right)$ in patients with non-ischemic cardiomyopathy showed results similar to datasets of healthy subjects, but the coefficient of determination (*R*^2^ = 0.20) was notably smaller than in healthy subjects. To compare QTV of healthy subjects to that of DEFINITE patients, the TQT dataset has been used. As QT recordings started approximately at 7 o'clock (07:08 ± 00:26 h), segments between 7 and 8 o'clock of the DEFINITE data have been used to minimize the influence of circadian rhythm (Schmidt et al., 2014a). Note that we could not take into account individual differences in circadian rhythmicity (i.e., chronotypes) and individual activities because no time resolved individual information on the patient state was available. A random lead selection was applied to the TQT dataset (12-Lead standard ECG) for comparison to the analyzed lead Z of the DEFINITE data. We found smaller ${\bar{T}}_{amp}$ in TQT (74.35 ± 58.30 μ*V*) compared to DEFINITE (187.18 ± 158.05 μ*V*). SDQT showed significant differences [*p* < 0.005 (Student's *t*-test)] between TQT (13.46 ± 8.81 *ms*) and DEFINITE (16.73 ± 10.41 *ms*). Similarly, cSDQT showed highly significant differences (TQT: 6.60 ± 2.77 *ms*; DEFINITE: 12.47 ± 7.36 *ms*; *p* < 0.001). However, Cohen's d calculated between both groups increased 2.5-times (from 0.31 to 0.80; Figure 2).

**Figure 3:**
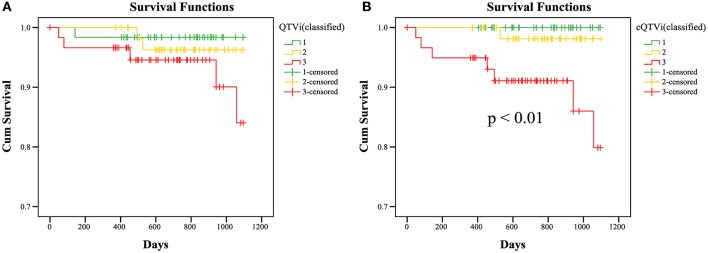
**KaplanMeier survival curves; Estimated survival of DEFINITE patients for QTVi (A) and cQTVi (B; classified in tertiles: 1 – lowest tertile, 2 – medium tertile, 3 – highest tertile) for 3 years**. Last observations of a patient are marked in the curves with a cross (censored data).

To assess the predictive value of QTV Kaplan-Meier survival curves were generated for trichotomized corrected QTVi (cQTVi) values (1 – lowest tertile, 2 – medium tertile, 3– highest tertile) of baseline Holter ECG. Results show significant association between survival and cQTVi (*p* < 0.01 by the log-rank test; Figure 3). At year one the rate of death was 0.0% in the low and the medium cQTVi group and 5.1% in the group with high cQTVi. After 2 years, it was 0.0% in the low cQTVi group, 1.9% in the medium cQTVi group and 8.9% in the group with high cQTVi. At the end of survival estimation (after 3 years) cQTVi was 0.0 in the low cQTVi group, 1.9% in the medium cQTVi group and 20.1% in the group with high cQTVi. We found qualitatively similar results when using uncorrected QTVi, but the difference in survival across tertiles was not statistically significant (*p* = 0.12; Figure 3).

## 4. Discussion

In this study we have introduced a novel T wave amplitude correction formula for QTV analysis. Its potential for improving QTV assessment was demonstrated by using QTV as predictor for all-cause mortality in heart failure patients (DEFINITE; *p* < 0.01). Without correction, QTV prediction of all-cause mortality is not significant (*p* = 0.12). At the same time, expected effects of the sympathetic nervous system on QTV can be equally shown even after correction (ATHLETE). Both are considered important applications of QTV analysis (Baumert et al., 2016a).

In previous studies (Baumert et al., 2012; Hasan et al., 2012; Schmidt et al., 2014b) as well as the current one smaller T wave amplitudes have been shown to artificially increase QTV owing to the noise sensitivity of common QT interval extraction techniques. To minimize the influence of T wave amplitude, we propose a simple correction of QTV parameters for T wave amplitude. The correction was devised based on three datasets of healthy subjects that were independently acquired under different conditions, using different equipment, hence providing a representative sample of ECG recordings. Linear regression analyses of double log-transformed data have shown similar behavior in all three datasets that confirm the findings of Hasan et al. (2012). The choices of QT interval extraction algorithm and ECG lead have not shown any major effect on the relation between T wave amplitude and QTV.

To adjust common QTV metrics for T wave amplitude dependency, we introduced a correction formula that yields T wave amplitude corrected standard deviation of QT intervals (cSDQT), offering the possibility for QTV measurement that is less sensitive to T waveform characteristics. Furthermore, by normalizing QTV to a specific T wave amplitude, comparisons of QTV between leads or groups of different T wave amplitudes become more meaningful. By adjusting the calculation of the frequently used QTVi metric accordingly, a more precise non-invasive measure of repolarization lability is obtained. Applying the correction formula to QTV measurements is straight forward; the only additional data needed is the median T wave amplitude. In this regard, the 2DSW algorithm, for example, offers the possibility to extract both, QT interval and T wave amplitude.

Higher QTV has been previously reported in normal subjects in conditions of sympathetic activation such as standing, and hence it has been argued that high QTV is indicative of high sympathetic tone. Given that T wave amplitude during standing is smaller than in the supine position, previous observations might have at least in part been the result of the inverse T wave amplitude QTV relationship. Our results demonstrate for the first time that the difference in QTV observed between standing and lying is not solely caused by the reduction in T wave amplitude.

When exploring the T wave amplitude—QTV relationship in a clinical dataset of Holter ECG, we found a regression coefficient similar to that of healthy subjects but a smaller *R*^2^. Pathologic cardiac repolarization in patients with non-ischemic cardiomyopathy might partly explain the increased variance in the dataset. When comparing QTV between patients enrolled in the DEFINITE study and healthy subjects, applying the T wave amplitude correction achieves 2.5-times better group discrimination by minimizing the inter-group variability of T wave amplitude influence. Kaplan-Meier estimator analysis showed that cQTVi is inversely related to survival. Patients with non-ischemic cardiomyopathy and a high cQTVi have a significant higher all-cause mortality than patients with low cQTVi. Importantly, QTV was only predictive of all-cause mortality after correcting for T wave amplitude.

Previous studies have repeatedly shown predictive value of QTV for mortality in patients with ischemic heart disease, where the mode of death is likely malignant ventricular arrhythmia (Baumert et al., 2016a), but its potential for risk stratification in patients with non-ischemic cardiomyopathy remains to be established.

In conclusion, all ECG datasets have shown a significant inverse relationship between T wave amplitude and QTV of similar characteristic. Exploiting this property, a simple correction formula has been proposed and improved QTV assessment for predicting all-cause mortality in patients with non-ischemic cardiomyopathy has been demonstrated.

## 5. Limitations

A potential limitation of the proposed correction formula is its dependence on the algorithm that is used to delineate the T wave. In principle, each algorithm might behave different as a function of T wave amplitude. To account for algorithm specific differences we did not rely on a single algorithm but invoked two algorithms for QT extraction. Both of them were previously shown to be very robust and appropriate for QTV measurement (Baumert et al., 2012; Schmidt et al., 2014b). The similar findings for both algorithm, not only qualitatively but also quantitatively, accounts for the general usability of the proposed correction.

Another potential limitation of the proposed algorithm is imposed by pathophysiologic alterations of T wave amplitude. QTV analysis is usually based on single ECG lead, representing the scalar projection of the cardiac electric vector on a particular point of the body surface. While in general those ECG leads are chosen that yield high T waves (Baumert et al., 2016a), e.g., lead II, and thereby a good signal-to-noise ratio, T wave axes may vary between individuals and hence may represent repolarization waves sub-optimally. This is particularly relevant in patients with cardiac myopathies where depolarization propagation and repolarization across the ventricular myocardium are highly individual. Hence, the predictive value of QTVi may partly lie in capturing differences in spatial axis-related patterns for the QRS and T waves. The T wave amplitude correction coefficient introduced in this paper is agnostic to the source underlying the T wave amplitude change. Regarding the lead which actually has been used in this study, it should be noted that not a single one was used, i.e., in parts differing leads have been used for the different studies. This is mainly a consequence of our aim to invoke as comprehensive a data set as possible. Owing to the availability of data and the retrospective nature of our study, we could not restrict ourselves to use the same lead. However, extensive pretests showed that, from an algorithmic point of view, behavior is similar across leads. Even the normalization to a “standard” T wave amplitude, which comes along with the proposed method, contributes to such comparability.

Finally we have to mention a limitation, which is relevant for future studies on QTVi, normative values of QTVi and its extended use as marker for risk stratification. Though the primary aim of the proposed correction is to account for the influence of T wave amplitude on QT variability metrics, as stated before our algorithm also normalizes the resulting QTV to a reference T wave amplitude. In principle, such normalization is desirable, as it improves comparability of QTV measurements between subjects and across studies. However, it should be noted that two different definitions of QTVi exist: the original formula relates QTV to heart rate variability (Berger et al., 1997), while a later modification uses RR variability instead (Piccirillo et al., 2001), and has been used in this study. Both definitions are currently in use and the lack of consensus adds another layer of complexity to the comparability of QTVi measurements. Importantly, the amplitude correction proposed in this paper is independent from the definition of QTVi and resulted in similar improvements for either definition (results on using HRV for normalization are not shown).

## Author contributions

MS processed ECG data, computed variability measures, conducted statistical analysis and drafted the manuscript. MB and SZ conducted statistical analysis and drafted the manuscript. HM drafted the manuscript. All authors have read and approved the final manuscript.

## Funding

This study was partly supported by grants from the Australian Research Council (DP 110102049), Group of Eight Australia and the German academic exchange service (DAAD).

### Conflict of interest statement

The authors declare that the research was conducted in the absence of any commercial or financial relationships that could be construed as a potential conflict of interest.
